# Antifungal lock therapy: an eternal promise or an effective alternative therapeutic approach?

**DOI:** 10.1111/lam.13653

**Published:** 2022-01-30

**Authors:** R. Kovács, L. Majoros

**Affiliations:** ^1^ 37599 Department of Medical Microbiology Faculty of Medicine University of Debrecen Debrecen Hungary; ^2^ 37599 Faculty of Pharmacy University of Debrecen Debrecen Hungary

**Keywords:** antifungal lock therapy, biofilm, *Candida*, *Candida auris*, candidaemia, catheter‐associated infection

## Abstract

Each year, millions of central venous catheter insertions are performed in intensive care units worldwide. The usage of these indwelling devices is associated with a high risk of bacterial and fungal colonization, leading to the development of microbial consortia, namely biofilms. These sessile structures provide fungal cells with resistance to the majority of antifungals, environmental stress and host immune responses. Based on different guidelines, colonized/infected catheters should be removed and changed immediately in the case of *Candida*‐related central line infections. However, catheter replacement is not feasible for all patient populations. An alternative therapeutic approach may be antifungal lock therapy, which has received high interest, especially in the last decade. This review summarizes the published *Candida*‐related *in vitro*, *in vivo* data and case studies in terms of antifungal lock therapy. The number of clinical studies remains limited and further studies are needed for safe implementation of the antifungal lock therapy into clinical practice.

## Graphical abstract

**Significance and impact of the study:** Antifungal lock therapy is a promising alternative therapeutic approach for the treatment of *Candida*‐related central line infections without catheter removal. This review summarizes the most relevant *in vitro*, *in vivo* and clinical data, published in the last two decades, regarded as antifungal lock therapy.

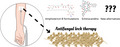

## Introduction

Based on the last published data, an estimated 5 million central venous catheters (CVCs) are inserted in patients annually in the United States alone, associated with nearly 250 000 bloodstream infections (O’Grady *et al*. 2011). Central line‐associated bloodstream infections (CLABSIs) are defined as a central line‐related laboratory‐confirmed bloodstream infection occurring more than 48 h following central line placement (Haddadin *et al*. 2021). The CLABSIs pose a serious problem to healthcare systems as they usually require a complex therapeutic approach and a prolonged length of hospital stay (13·13 ± 9·53 days) (Alotaibi *et al*. 2020). The International Nosocomial Infection Control Consortium (INICC) surveyed 703 intensive care units from 50 countries between 2010 and 2015; the CLABSI rate was 4·1 per 1000 central line days (Rosenthal *et al*. 2016). Mortality rates for diagnosed CLABSIs range from 12 to 25%, according to the National Healthcare Safety Network (Norris *et al*. 2017). It is noteworthy that the CLABSI rates increased by 51% during the first 6 months of the coronavirus disease 2019 (COVID‐19) pandemic compared to the 12 months prior at 78 hospitals in the United States (Fakih *et al*. 2021). Although most episodes are preventable with appropriately applied aseptic techniques, continuous surveillance and local hospital‐specific therapeutic strategies, increased incidences have been reported worldwide with regard to CLABSIs (Haddadin *et al*. 2021). In addition, the widespread use of intravascular devices, such as central venous and haemodialysis catheters, maintains this worrisome trend. The number of reported CLABSI in non‐intensive care units is variable ranging from 0·9 infections per 1,000 central‐line days to 5·2 episodes per 1000 catheter days (Rhee *et al*. 2015). In the past decade, an important epidemiological trend was noticed where the non‐intensive care unit associated CLABSI rate increased compared to concurrent intensive care unit rate (Tedja *et al*. 2014). Notably, a considerable proportion of CLABSI patients (70 out of 104 patients) outside the intensive care unit had underlying hematologic malignancy and nearly 92% of patients were neutropenic (Rhee *et al*. 2015).

Based on the epidemiological data derived from the past decade, *Candida* species are the fourth and seventh leading cause of bloodstream infections in the United States and Europe, respectively (Wisplinghoff *et al*. 2004). Additionally, the current incidence of candidaemia is significantly higher among COVID‐19‐infected patients compared to the non‐COVID‐19 group (Kayaaslan *et al*. 2021). *Candida*‐related bloodstream infections are associated with the highest overall mortality of all nosocomial bloodstream infections in intensive care units, ranging from 30 to 60% depending on the species, which are comparable to that of *Pseudomonas aeruginosa* (Wisplinghoff *et al*. 2004). Data from the centres for disease control and prevention (CDC) revealed that most pathogen‐specific CLABSI rates decreased over time in acute care hospitals between 2011 and 2017, except *Candida* species in adult intensive care units (Novosad *et al*. 2020). Based on this comprehensive study, these pathogens were the most common CLABSI‐related microorganisms in 2017 (Novosad *et al*. 2020).

*Candida* cells can adhere to the surface of indwelling devices and produce an extensive extracellular polysaccharide matrix that will further facilitate adhesion, thickening the developed biofilm (Cavalheiro and Teixeira 2018). In such a sessile community, *Candida* cells display increased resistance to traditional antifungal agents; in addition, cells may disperse from these sources into the bloodstream, leading to a life‐threatening infection (Cavalheiro and Teixeira, 2018; Vitális *et al*. 2020). Based on recent guidelines, immediate catheter replacement must be applied in the case of proven *Candida*‐related catheter infections (Mermel *et al*. 2009; O'Grady *et al*. 2011). However, changing the CVC may be complicated; furthermore, the exchange significantly increases the risk of bloodstream infection and complications, depending on the catheter location (eg, pneumothorax, arterial puncture) (O'Grady *et al*. 2011). In addition, catheter replacement is not feasible for all patients, such as premature neonates or critically ill patients who may be thrombocytopenic, coagulopathic or have limited venous access (Walraven and Lee 2013).

Several randomized clinical trials proved that the use of extremely high local concentrations of antibacterial compounds (100–1000 times planktonic MIC) alone or in combination can eradicate the difficult‐to‐treat intraluminal sessile bacterial communities and can significantly reduce the risk of catheter‐associated bacterial infections (Justo and Bookstaver 2014). The ideal lock solution should possess several characteristics such as a relatively wide spectrum, the ability to penetrate biofilms, compatibility with anticoagulant, prolonged stability, low risk of adverse effects, the low potential of resistance and cost‐effectiveness (Justo and Bookstaver 2014). Based on the last Infectious Disease Society of America (IDSA) practice guideline for the management of intravascular catheter‐related infections, the lock therapy is recommended as adjunctive therapy specifically for catheter salvage in certain clinical situations where the catheter is not removed, especially in the case of coagulase‐negative staphylococci (Mermel *et al*. 2009). Despite these relatively widely used antibacterial approaches, there is, so far, no approved antifungal lock therapeutic protocol in clinical practice.

This review focuses on the *in vitro*, *in vivo* and clinical data of the antifungal lock therapy available in the literature to summarize the current status of this promising antifungal treatment approach.

## Traditional antifungal agents related *in vitro* results

Over the last decade, the number of *in vitro* studies dealing with potential antifungal lock solutions against *Candida* biofilms has steadily increased (Walraven and Lee 2013; Imbert and Rammaert 2018). Most studies have used a static 96‐well static microplate model with or without frequently used catheter material (eg, silicone, polyvinyl chloride, etc.). On the one hand, these results are easy to use and to interpret; however, on the other hand, several limitations can be observed, such as the static nature, the total lack of host response and cellular components and different culturing media, which may influence the observed antifungal effect. Moreover, the metabolic activity changes of biofilms following antifungal drug exposure do not correlate linearly with the living cell number (Ramage 2016).

In one of the earliest study, Kuhn *et al*. (2002) reported that lipid formulations of amphotericin B (4 mg l^−1^) and the echinocandins (4 mg l^−1^) have a remarkable anti‐biofilm effect against *C. albicans* (*n* = 2) and *C. parapsilosis* (*n* = 2), significantly higher than that of azoles (Katragkou *et al*. 2008). Miceli *et al*. (2009a) and Ku *et al*. (2011) demonstrated that anidulafungin (ANI), caspofungin (CAS) and micafungin (MICA) produced comparable high anti‐biofilm effects against *C. albicans* (SC5314 reference strain) and *C. tropicalis* (*n* = 5), using an XTT assay‐based static microplate model. The possible observed differences may be explained by the occurrence of paradoxical growth at high dosages, which was most significant in the case of CAS, followed by ANI (Simitsopoulou *et al*. 2013; Prażyńska and Gospodarek‐Komkowska 2019). Furthermore, the susceptibility of *Candida* biofilms to echinocandins may influence the metabolic activity of sessile cells. Marcos‐Zambrano *et al*. (2014) reported that MICA is more active against *C. albicans* biofilms (*n* = 265) with high metabolic activity, whereas the efficacy of CAS and ANI is not affected by the metabolic activity of biofilms (Marcos‐Zambrano *et al*. 2016). Regarding early studies, Cateau *et al*. (2008) examined the potential role of CAS (2 mg l^−1^) and MICA (5 mg l^−1^) as a lock solution against early (12 h) and mature (5 days) biofilms formed by *C. albicans* reference strains (*n* = 2), using sections of 100% silicone catheters. Biofilm growth inhibition was observed after the end of a 12‐h antifungal treatment period. The catheter was incubated without echinocandins for 24, 48 or 72 h after the lock therapy. Significant metabolic activity reduction was observed, irrespective of the maturation stage; moreover, this observed metabolic activity decrease was maintained even after 2 days (Cateau *et al*. 2008). Simitsopoulou *et al*. (2014) examined the activity of CAS at catheter lock concentration against two rarely isolated *Candida* species, *C. guilliermondii* (*n* = 5) and *C. lusitaniae* (*n* = 6). This study showed that CAS and L‐AMB had the highest activity against the tested biofilms, although the activity of L‐AMB was significantly lower than that exerted by CAS (512–2048 mg l^−1^) (Simitsopoulou *et al*. 2014). Kovács *et al*. (2019) reported that nikkomycin Z may be a possible adjuvant in lock therapy in combination with echinocandins. In these experiments, nikkomycin Z enhanced the activities of CAS and MICA against 1‐day‐old biofilms formed by echinocandin‐susceptible (*n* = 5) and echinocandin‐resistant *C. albicans* (*n* = 1) strains (Kovács *et al*. 2019).

Ko *et al*. (2010) examined the efficacy of 1 mg ml^−1^ amphotericin B deoxycholate (d‐AMB), CAS, fluconazole (FLU), itraconazole (ITRA) and voriconazole (VOR) against a one‐one clinical strain of *C. albicans*, *C. glabrata* and *C. tropicalis*, using a polyurethane segment colonization model. The biofilm killing patterns of antifungal agent tested were assayed after lock periods of 1, 3, 5, 7, 10 or 14 days, where the antifungal lock solutions were replaced every 2 days. Interestingly, the obtained results suggested that ITRA, FLU and VORI may be potential lock agents, whereas the efficacy of d‐AMB and CAS was questionable at the tested concentration (Ko *et al*. 2010). Öncü (2011) tested the potential use of d‐AMB, CAS, FLU, ITRA and VOR as a lock solution against one‐one clinical *C. albicans* and *C. parapsilosis* isolates, using the silicone catheter segment model. The examined concentrations were 300, 500 and 1000 times higher than the MIC values. Different drugs were assayed after lock periods of 1, 3, 5 and 7 days, where lock solutions were replaced every 2 days. The d‐AMB and CAS lock treatment showed complete inhibition, whereas FLU, ITRA and VOR had no effect (Öncü 2011), although the authors used azole‐based lock solutions at significantly lower concentrations. *C. albicans* (*n* = 8) and *C. glabrata* (*n* = 6) clinical isolates were examined against MICA, CAS and posaconazole (POSA) (10 mg l^−1^) lock solutions, using a silicone catheter segment model by Cateau *et al*. (2011). Lock efficacy was examined against early (12 h) and mature (5 days) biofilms following exposure to CAS (5 and 25 mg l^−1^), MICA (5 and 15 mg l^−1^) and POSA (10 mg l^−1^) for 12 h. The results showed that MICA had the highest inhibitory efficacy against early and mature *C. albicans* and *C. glabrata* biofilms. Moreover, this activity appeared to persist for up to 3 days. Toulet *et al*. (2012) tested the efficacy of L‐AMB‐based lock solution (200 and 1000 mg l^−1^) against early (12 h) and mature (5 days) biofilms formed by six clinical *C. albicans*, *C. glabrata* and *C. parapsilosis* isolates, using a silicon segment model. The lock solution at the highest concentration strongly inhibited early and mature biofilms for up to 48 h after the end of the lock. However, total eradication of the sessile cells was not obtained using 1000 mg l^−1^ L‐AMB as a single lock.

## Drug repurposing and alternative potential lock solutions

Miceli *et al*. (2009b) combined high‐dose doxycycline (128 512 and 2028 mg l^−1^) with different concentrations of FLU, CAS and d‐AMB (2–1024 mg l^−1^) and determined their antifungal efficacy against *C. albicans* SC5314 reference strain, using the XTT‐assay. It is noteworthy that doxycycline alone (at 2048 and 1024 mg l^−1^) demonstrated up to an 85% decrease of the metabolic activity of the *C. albicans* biofilm following 24 h of drug exposure. Doxycycline at 128 mg l^−1^ in combination with FLU demonstrated synergistic interaction. Furthermore, the combination of doxycycline at 2048 or 512 mg l^−1^ and d‐AMB was superior to d‐AMB alone at low concentrations (0·25–0·03 mg l^−1^). The same group examined the efficacy of pure heparin and its two preservatives methylparaben and propylparaben against *C. albicans* clinical isolates (*n* = 5) (Miceli and Chandrasekar 2012). Pure heparin, methylparaben and propylparaben caused up to 75, 85 and 60% decreases in the metabolic activity of mature *C. albicans* biofilms. Complete inhibition of biofilm formation was observed with a heparin sodium preparation at 5000 U ml^−1^ and higher. Furthermore, the authors demonstrated that the combination of high concentrations (5× or higher) of pure heparin, methylparaben and propylparaben in the proportions contained in heparin sodium solution had a synergistic effect on *C. albicans* SC5314 mature biofilms (Miceli and Chandrasekar 2012).

Tigecycline was tested by Ku *et al*. (2010) against SC5314 *C. albicans* biofilms; the data indicated that tigecycline at high doses is highly active *in vitro* against *C. albicans* biofilms. Tigecycline inhibited the formation of biofilms from 128 mg l^−1^. Against mature biofilms, 2048 mg l^−1^ tigecycline reduced metabolic activity by 84·2%. It could also enhance the activity of FLU and d‐AMB against sessile cells; however, the observed effect was not superior to that of 512 mg l^−1^ tigecycline alone, which could significantly inhibit (more than 50%) the metabolic activity of *C. albicans* biofilms (Ku *et al*. 2010). Regarding further antibiotics, the antibacterial drugs cefepime, meropenem, piperacillin/tazobactam and vancomycin, at concentrations from MIC/10 to 50 × MIC, decreased the *in vitro* viability of mature *C. albicans* (*n* = 10) and *C. tropicalis* (*n* = 10) biofilms (Sidrim *et al*. 2015).

Raad *et al*. (2008) determined the activity of the amphotericin B lipid complex (ABLC) (2 mg ml^−1^) in the presence or absence of EDTA (30 mg ml^−1^) against five‐five *C. albicans* and *C. parapsilosis* biofilms, using the silicon disk colonization model. The disks were incubated for 6 and 8 h in lock solutions containing ABLC alone, EDTA alone and ABLC in combination with EDTA. The ABLC with EDTA was significantly more effective compared to the compounds used alone against *C. parapsilosis* at 6 h and *C. albicans* at 8 h. Martins *et al*. (2012) reported an improved efficacy of d‐AMB when it was used in combination with DNase against *C. albicans* biofilm (SC5314 reference strain), using the XTT‐assay and quantitative culturing, whereas DNase treatment did not significantly enhance the effect of CAS and FLU. Bergamo *et al*. (2015) examined the potential activity of imidazolium salt (C_16_MImCl) against *C. tropicalis* biofilms (*n* = 6) formed by clinical isolates, using polyvinyl‐chloride catheter segments. The tested imidazolium salt prevented the biofilm formation of *C. tropicalis* in concentrations as low as 0·028 mg l^−1^. Similarly, cerium‐nitrate also exhibited a considerable antifungal effect against developed biofilms of various *Candida* species at concentrations from 16 to 1000 mmol l^−1^, resulting in a decrease in the biomass and metabolic activity of preformed sessile cells (Silva‐Dias *et al*. 2015). In these experiments, *C. glabrata* isolates (*n* = 8) showed the highest resistance to cerium‐nitrate treatment, whereas *C. guilliermondii* strains (*n* = 8) were the most susceptible ones (Silva‐Dias *et al*. 2015). Rosenblatt *et al*. (2013) examined the effect of glyceryl‐trinitrate in the presence of adjuvant compounds against bacterial and fungal biofilms to test for the eradication of biofilm within 2 h of lock exposure on low‐surface‐energy silicone rubber surfaces. The authors reported that the addition of 7% citrate to 0·1% glyceryl‐trinitrate plus 6% ethanol plus 6% propylene glycol could totally eradicate biofilm formed by *C. albicans* (*n* = 1), showing synergy for this organism. *In vitro* data published by Alonso *et al*. (2018) revealed that an ethanol‐based lock solution with 40% ethanol + 60 IU heparin, administered daily for 72 h, is sufficient to almost eradicate the metabolic activity of biofilms formed by a *C. albicans* reference strain. Based on the published data, heparin is a frequently used component of promising lock solutions. However, the addition of heparin to a lock solution has two major disadvantages. First, we have to consider the potential emergence of allergy‐ and heparin‐associated thrombocytopenia, which may occur in 10–30% of patients receiving heparin (Shantsila *et al*. 2009). Second, heparin promotes the biofilm formation of *Candida* cells (Green *et al*. 2013). Reitzel *et al*. (2016) compared the efficacy of nitroglycerin‐citrate‐ethanol to (0·0015 to 0·003% nitroglycerine—4% citrate–22% EtOH) to 1·35% taurolidine–3·5% citrate—1000 U ml^−1^ heparin. The tested nitroglycerin‐citrate‐ethanol was superior to taurolidine‐citrate‐heparin against *C. glabrata* biofilm (*n* = 1) (Reitzel *et al*. 2016). A recent study demonstrated the potential usage of aspirin as a lock component (Chan *et al*. 2021). In this study, *C. albicans* (*n* = 1) and *C. tropicalis* (*n* = 1) biofilms were most sensitive to aspirin exposure. The *C. albicans* biofilm was eradicated by aspirin at a concentration of 40 mg ml^−1^ in 4 h. Moreover, *C. glabrata* (*n* = 1) *C. krusei* (*n* = 1) and *C*. *tropicalis* biofilms were eradicated by aspirin at a concentration of 40 mg ml^−1^ in 24 h (Chan *et al*. 2021).

Based on *in vitro* data, ethanol‐based lock solutions appear as some of the most promising antifungal lock strategies; however, more limitations may emerge in terms of the usage of this compound, such as its potential incompatibility with polyurethane catheters. Nonetheless, Raad *et al*. (2007) compared minocycline, EDTA and 25% ethanol against one *C. parapsilosis* strain, using silicone catheter segments. Both ethanol‐EDTA and ethanol‐EDTA‐minocycline combinations eradicated 100% of the growth of the *C. parapsilosis* biofilm when tested for 1 day. Based on the results published by Balestrino *et al*. (2009), 60% ethanol eliminated *C. albicans* SC5314 biofilm from silicone catheter segments following 20 min of exposure. Venkatesh *et al*. (2009) found that 12·5% ethanol treatment significantly reduced the metabolic activity and living cell number of *C. albicans* biofilms. In addition, this ethanol concentration showed a synergistic interaction with d‐AMB and FLU. Ghannoum *et al*. (2011) performed a comprehensive study in which 5 mg ml^−1^ trimethropim, 25% ethanol and 3% EDTA were combined, inhibiting all *Candida* isolates examined, namely *C. albicans* (*n* = 25), *C. glabrata* (*n* = 25), *C. tropicalis* (*n* = 25) and *C. krusei* (*n* = 25). A three component‐based lock solution was tested by Lown *et al*. (2016). The solution containing 20% (v/v) ethanol, 0·01565 mg l^−1^ MICA and 800 mg l^−1^ doxycycline demonstrated a 98% reduction in metabolic activity; however, there was no advantage over 20% ethanol alone (Lown *et al*. 2016).

A relatively novel and innovative lock strategy is the usage of gas plasma‐activated disinfectants (Bhatt *et al*. 2018). It is noteworthy that viable cells of *C. albicans* in mature biofilms decreased by 6–8 orders of magnitude with a novel antifungal‐free lock solution formed from gas plasma‐activated disinfectant for 60 min. For comparison, the usage of a minocycline‐EDTA‐ethanol lock solution for 60 min resulted in a significant regrowth of fungal cells within 24 h (Bhatt *et al*. 2018).

Eventually, the steadily expanding list of natural substances with potential anti‐*Candida* activity has to be highlighted. Nearly 150 natural products have a remarkable antifungal effect, which may be potential drug(s) and/or adjuvant(s) in lock therapy in the future. Nonetheless, several investigations must be pursued to give more details with regard to molecule structure, activity and interaction to apply them safely (Zida *et al*. 2017; Donadu *et al*. 2020, 2021).

## Promising antifungal lock solutions against *Candida auris in vitro*

*Candida auris* is posing a continuous global public health threat due to its ability to cause nosocomial outbreaks of invasive infections in healthcare environments worldwide (Du *et al*. 2020). The biofilm‐forming ability of this species and its role in catheter‐associated infections are known phenomena (Horton and Nett 2020; Sayeed *et al*. 2020). However, the treatment of this central line infection is a particularly hard task due to the multiresistant phenotype of *C. auris*, justifying this separate section within this review. Recently, several studies focusing on potential lock solutions against *C. auris* have been published. Vargas‐Cruz *et al*. (2019) compared the efficacy of traditional antifungal agents to nitroglycerine‐citrate‐ethanol catheter lock solution. Testing was performed on 1‐day‐old *C. auris* biofilms followed by 2 h of exposure to the lock solutions. In their study, L‐AMB (1 mg ml^−1^), d‐AMB (0·1 mg ml^−1^), FLU (2 mg ml^−1^), VOR (0·5 mg ml^−1^), MICA (0·5 mg ml^−1^), CAS (0·5 mg ml^−1^) and ANI (0·5 mg ml^−1^) failed to completely eradicate all 10 tested *C. auris* biofilms (Vargas‐Cruz *et al*. 2019). Conversely, nitroglycerine (0·003%), citrate (4%), ethanol (22%) lock solution completely eradicated all *C. auris* biofilms examined (Vargas‐Cruz *et al*. 2019). Reitzel *et al*. (2020) examined the effect of aminocycline (0·1%) ‐EDTA (3%) ‐ethanol (25%) lock against *C. auris* biofilms. This three‐component solution was able to fully eradicate all 10 tested isolates of *C. auris* biofilms following 60 min of exposure (Reitzel *et al*. 2020). A promising alternative therapeutic approach is the treatment disrupting quorum‐sensing by the usage of quorum‐sensing molecules at supraphysiological concentrations, which may enhance the activity of traditional antifungal agents (Kovács and Majoros 2020). Regarding *C. auris* biofilms, Nagy *et al*. (2020a, 2020b) examined the effects of echinocandins and triazole in the presence of farnesol as potential lock solutions against 1‐day‐old *C. auris* biofilms (*n* = 7). According to the FIC index determination, farnesol could significantly enhance the activities of ANI, CAS and MICA (FICI range 0·133–0·5). Additionally, the interaction between FLU, ITRA, VOR, POSA, isavuconazole (ISA) and farnesol showed clear synergism (FICI range from 0·038 to 0·375) against 1‐day‐old biofilms. A further alternative lock solution contained small cysteine‐rich cationic antifungal protein, *Neosartorya fischeri* antifungal protein 2 (NFAP2), which exerted synergistic interactions with FLU, AMB, ANI, CAS and MICA, with FICIs ranging between 0·064 and 0·5 against *C. auris* biofilms (*n* = 5). Moreover, sessile cells exposed to three echinocandins (32 mg l^−1^) exhibited significant cell death in the presence of NFAP2 (128 mg l^−1^) (Kovács *et al*. 2021).

## *In vivo* studies of antifungal lock strategies

The number of valid *in vivo* experiments dealing with potential antifungal lock solutions is strongly limited (Table 1). Most studies tested various formulations of AMB alone or in combination with another traditional antifungal drug. In one of the earliest studies, Mukherjee *et al*. (2009) used a rabbit CVC model of *C. albicans* to examine the efficacy of ABLC (5 mg ml^−1^ for 4 and 8 h per day, respectively). On day 11 of the lock treatment, both arms completely sterilized the ABLC‐locked catheters (Mukherjee *et al*. 2009). In another study, Schinabeck *et al*. (2004) compared the efficacy of L‐AMB (10 mg ml^−1^) with that of FLU (10 mg ml^−1^) against *C. albicans* using a rabbit CVC model, where the L‐AMB lock solution could completely sterilize the catheters. Shuford *et al*. (2006) observed a superior effect of CAS lock (6·67 mg ml^−1^) compared to d‐AMB (3·33 mg ml^−1^) in a 7‐day lock model of a *C. albicans* catheter infection. Fujimoto and Takemoto (2018) compared the *in vivo* activity of 2 mg l^−1^ L‐AMB lock to that of 2 mg l^−1^ MICA lock solution against *C. albicans*, *C. glabrata*, *C. parapsilosis* and *C. tropicalis*. The L‐AMB lock showed a superior effect against *C. parapsilosis* using the murine CVC model; however, the efficacy of the two tested lock solutions was comparable to those of *C. albicans*, *C. glabrata* and *C. tropicalis* (Fujimoto and Takemoto 2018). In another study, ANI (3·3 mg ml^−1^) exerted a significant decrease relative to L‐AMB (5·5 mg ml^−1^) for *C. parapsilosis* isolates *in vivo*. In addition, only ANI achieved negative catheter tip cultures (Basas *et al*. 2016). In the case of *C. albicans*, both L‐AMB (5 mg ml^−1^) and ANI (3·3 mg ml^−1^) produced significant reductions compared to growth control recovered from the catheter tips, whereas ANI lock solution achieved significant reductions compared to other treatments in the case of *C. glabrata* (Basas *et al*. 2019). Lazzell *et al*. (2009) examined the efficacy of CAS (0·25 mg l^−1^) lock for the treatment and prevention of biofilms formed by a *C. albicans* reference strain. The 24‐h‐long lock time significantly reduced the fungal burden derived from catheters when used for either treatment or prevention in a murine CVC model (Lazzell *et al*. 2009). A comparable therapeutic success was observed in another study (Salinas *et al*. 2019), where a 16‐mg l^−1^ daily lock treatment was examined with 1 mg kg^−1^ daily systemic MICA therapy. The treatment started on day 1, lasted for 7 days and was followed by 7 days of surveillance without treatment. In this study, the therapeutic success was 75% compared to the results reported by Lazzel *et al*. (2009), who observed 67% (Salinas *et al*. 2019).

**Table 1:** *In vivo* result of antifungal lock therapy against various *Candida* species

Reference	*Candida* species/strain	Animal/Model	Lock solution	Duration of therapy	Therapeutic success
Schinabeck *et al*. (2004)	*C. albicans* (M61 strain)	Rabbit CVC model	10 mg ml^−1^ L‐AMB	8 h per day for 7 days	7/7 (100%)
			10 mg ml^−1^ FLU		2/7 (29%)
Shuford *et al*. (2006)	*C. albicans* (IDRL‐5319)	Rabbit CVC model	6·67 mg ml^−1^ CAS	7 days	16/16 (100%)
			3·33 mg ml^−1^ d‐AMD		13/16 (81%)
Mukherjee *et al*. (2009)	*C. albicans* (M61 strain)	Rabbit CVC model	5 mg ml^−1^ ABLC	4 h per day for 7 days	6/6 (100%)
			5 mg ml^−1^ ABLC	8 h per day for 7 days	6/6 (100%)
Lazell *et al*. (2009)	*C. albicans* (SC5314 strain)	Murine CVC model	0·25 mg l^−1^ CAS	24 h	4/6 (67%)
Basas *et al*. (2016)	*C. parapsilosis* (CP12 strain)	Rabbit CVC model	5·5 mg ml^−1^ L‐AMB	48 h	3/10 (30%)
			3·3 mg ml^−1^ ANI		5/8 (63%)
	*C. parapsilosis* (CP54 strain)		5·5 mg ml^−1^ L‐AMB		1/6 (17%)
			3·3 mg ml^−1^ ANI		8/11 (73%)
Fujimoto and Takemoto (2018)	*C. albicans* (SP‐20012)	Murine CVC model	2 mg l^−1^ daily lock L‐AMB + 5 mg kg^−1^ daily i.p. L‐AMB	72 h	7/7 (100%)
			2 mg l^−1^ daily lock MICA + 15 mg kg^−1^ daily i.p. MICA		7/7 (100%)
	*C. glabrata* (SP‐20040)		2 mg l^−1^ daily lock L‐AMB + 5 mg kg^−1^ daily i.p. L‐AMB		8/7 (88%)
			2 mg l^−1^ daily lock MICA + 15 mg kg^−1^ daily i.p. MICA		8/8 (100%)
	*C. glabrata* (SP‐20040)		2 mg l^−1^ daily lock L‐AMB + 5 mg kg^−1^ daily i.p. L‐AMB		6/5 (83%)
			2 mg l^−1^ daily lock MICA + 15 mg kg^−1^ daily i.p. MICA		6/4 (67%)
	*C. parapsilosis* (SP‐20137)		2 mg l^−1^ daily lock L‐AMB + 5 mg kg^−1^ daily i.p. L‐AMB		8/7 (88%)
			2 mg l^−1^ daily lock MICA + 15 mg kg^−1^ daily i.p. MICA		8/4 (50%)
	*C. tropicalis* (SP‐20047)		2 mg l^−1^ daily lock L‐AMB + 5 mg kg^−1^ daily i.p. L‐AMB		6/6 (100%)
			2 mg l^−1^ daily lock MICA + 15 mg kg^−1^ daily i.p. MICA		6/6 (100%)
Salinas *et al*. (2019)	*C. albicans* (SKCA23‐ACTgLuc)	Murine CVC model	16 mg l^−1^ daily lock MICA + 1 mg kg^−1^ daily i.p. MICA	Treatment started at day 1, lasted 7 days, and was followed by 7 days of surveillance with no treatment	8/6 (75%)
Basas *et al*. (2019)	*C. albicans* (CA176)	Rabbit CVC model	5 mg ml^−1^ L‐AMB	48 h	10/5 (50%)
			3·33 mg ml^−1^ ANI		10/4 (40%)
	*C. albicans* (CA180)		5 mg ml^−1^ L‐AMB		6/5 (83%)
			3·33 mg ml^−1^ ANI		6/5 (83%)
	*C. glabrata* (CG171)		5 mg ml^−1^ L‐AMB		14/3 (21%)
			3·33 mg ml^−1^ ANI		11/7 (64%)
	*C. glabrata* (CG334)		5 mg ml^−1^ L‐AMB		7/2 (29%)
			3·33 mg ml^−1^ ANI		8/8 (100%)

## Clinical studies of antifungal lock therapies

The available case studies with regard to antifungal lock therapies against *Candida* species are summarized in Table 2. Most case studies dealing with antifungal lock therapies focused on paediatric patients, and the number of adult‐related studies is strongly limited (Table 2). Regarding the traditional antifungal agents, until recently, the AMB‐based lock solutions were the most frequently tested compounds. Overall, d‐AMB locks (2–2·5 mg ml^−1^) produced an 89% therapeutic success (100, 75 and 100% therapeutic success for *C. albicans*, C. glabrata and *C. parapsilosis*, respectively). The L‐AMB showed efficacy comparable to that of d‐AMB, where 83% overall therapeutic success was reported (75, 100 and 100% for *C. albicans*, *C. glabrata* and *C. guilliermondii*, respectively) (Table 2). Two reports of an echinocandin lock mono‐therapy can be found in the literature (Özdemir *et al*. 2011; Isgüder *et al*. 2017). In these cases, CAS lock solution was used with systemic CAS treatment for the treatment of *Candida lypolitica* catheter‐related bloodstream infections (Özdemir *et al*. 2011). The lock used a portion of a 3 ml solution of 10 mg CAS and 5% dextrose with 200 units of heparin in lines for 12 h per day for 14 days. The obtained cultures were negative after day 4 of treatment (Özdemir *et al*. 2011). Isgüder *et al*. (2017) followed the previous lock protocol for the treatment of a catheter‐related bloodstream infection caused by *C. parapsilosis*; however, the therapy was unsuccessful. Regarding alternative lock compounds, Blackwood *et al*. (2011) reported 100% lock efficacy of a heparin‐free 70% ethanol in three paediatric patients against two *C. albicans* isolates and one *C. parapsilosis* isolate. In a case study published by Piersigilli *et al*. (2014), systemic antifungal treatment (5 mg kg^−1^ L‐AMB) did not resolve the candidaemia. Lock therapy with 70% ethanol combined with 5 mg l^−1^ MICA was added to the therapy, which resulted in sterile blood cultures (Piersigilli *et al*. 2014). Recently, a total of 123 ethanol lock therapy episodes among 95 patients were analysed (including *Mycobacterium*, *Staphylococcus aureus*, *Candida* episodes) (Ashkenazi‐Hoffnung *et al*. 2021). Overall, successful catheter salvage was observed in 78% compared to episodes where systemic antimicrobials were used alone (54%). Multivariate analysis revealed four major predisposing factors in the case of ethanol lock therapy failure, such as the presence of Gram‐positive bacteria, elevated C‐reactive protein, signs of tunnel infection, low neutrophil count (Ashkenazi‐Hoffnung *et al*. 2021). In addition, the usage of ethanol as a lock solution may be associated with potential risks. Ethanol locks with concentrations above 28–30% have been related to clotting, dizziness, protein precipitation and compromised catheter integrity in polyurethane catheters (Mermel and Alang 2014; Schiller *et al*. 2014).

**Table 2:** Case reports of antifungal lock therapy against *Candida* species

Reference	Patient(s)	Species	Lock solution	Systemic therapy	Duration of therapy	Therapeutic success
Johnson *et al*. (1994)	4‐yr‐old sex is unknown	*C. albicans*	2 mg ml^−1^ d‐AMB	Unknown	12 h twice a day for 10–14 days	2/2 (100%)
	18‐yr‐old sex is unknown	*C. albicans*	2 mg ml^−1^ d‐AMB	Unknown	12 h twice a day for 10–14 days	
Benoit *et al*. (1995)	30‐yr‐old female	*C. glabrata*	2·5 mg ml^−1^ d‐AMB	d‐AMB for three days then FLU for 4 days	8–12 h/day for 15 days	2/1 (50%)
	40‐yr‐old female	*C. glabrata*	2·5 mg ml^−1^ d‐AMB	FLU for three days	6 h per day for 14 days	
		*C. albicans*	2·5 mg ml^−1^ d‐AMB	d‐AMB for 1 day	6 h per day for 14 days	
Viale *et al*. (2001)	2‐yr‐old female	*C. albicans*	2·5 mg ml^−1^ d‐AMB	d‐AMB for 7 days	12 h per day for 14 days	2/2 (100%)
	65‐yr‐old male	*C. albicans*	2·5 mg ml^−1^ d‐AMB	FLU for 7 days	12 h per day for 14 days	
Castagnola *et al*. (2005)	Infant	*C. parapsilosis*	2·67 mg ml^−1^ L‐AMB	L‐AMB for 14 days	8 h per day for 14 days	1/1 (100%)
Angel‐Moreno *et al*. (2005)	40‐yr‐old male	*C. glabrata*	5 mg ml^−1^ d‐AMB	Systemic FLU (no duration and dosage)	6 h per day for 14 days	1/1 (100%)
Wu and Lee (2007)	13‐yr‐old female	*C. parapsilosis*	2·5 mg ml^−1^ d‐AMB	d‐AMB for 6 days then FLU (no duration and dosage)	24 h per day for 20 days	1/1 (100%)
Buckler *et al*. (2008)	17‐mo‐old female	*C. albicans*	2·67 mg ml^−1^ L‐AMB	Systemic FLU for 5 days, which was changed to L‐AMB	8 h per day for 7 to 16 days	4/2 (50%)
		*C. glabrata*				
	7‐yr‐old female	*C. albicans*		Systemic L‐AMB	8 h per day for 17 days	
	6‐mo‐old male	*C. parapsilosis*			8 h per day for 15 days	
	1‐yr‐old female	*C. guilliermondii*			8 h per day for 14 days	
Özdemir *et al*. (2011)	9‐yr‐old male	*C. lypolitica*	3·3 mg ml^−1^ CAS	CAS + meropenem and teicoplanin	12 h per day for 14 days	1/1 (100%)
Blackwood *et al*. (2011)	8‐mo‐old male	*C. albicans*	70% ethanol	Systemic FLU	14 days	3/3 (100%)
	8‐mo‐old female	*C. parapsilosis*		Systemic VOR		
	5‐yr‐old male	*C. albicans*		Systemic FLU		
Paul DiMondi *et al*. (2014)	64‐yr‐old female	*C. albicans*	2·67 mg ml^−1^ L‐AMB	MICA for 14 days	24 h per day, change every 12 h, for 6 days	1/1 (100%)
Piersigilli *et al*. (2014)	Infant male	*C. albicans*	70% ethanol combined with 5 mg l^−1^ MICA	5 mg kg^−1^ L‐AMB then 10 mg kg^−1^ MICA	12 h	1/1 (100%)
Isgüder *et al*. (2017)	1·5‐yr‐old male	*C. parapsilosis*	3·33 mg ml^−1^ CAS	Systemic CAS	12 h per day for 14 days	1/0 (0%)

## Conclusions

Antibiotic lock strategies have been introduced in clinical practice in the late 1980s and have been recommended for the prevention and treatment of certain catheter‐associated infections by the IDSA and the CDC. Until recently, there was no officially approved antifungal lock therapeutic strategy, despite the fact that the majority of *Candida* bloodstream infections in long‐term CVCs are associated with a prominent intraluminal *Candida* colonization, serving as a continuous source of life‐threatening candidaemia. Based on the available *in vitro*, *in vivo* and clinical data, the ethanol‐based lock solutions show the highest activity. Nevertheless, the ethanol‐associated risks and limiting factors are well‐defined, impeding its widespread clinical use. There are *in vitro* data regarding echinocandin‐ and amphotericin B‐based lock solution alone or in combination with promising adjunctive compounds with various mechanisms; however, the number of valid *in vivo* studies dealing with these combinations is scarce. In summary, the forthcoming introduction of antifungal lock therapy into clinical practice remains questionable because several randomized clinical studies of the most promising combinations are needed to assess the clinical safety and efficacy of these promising strategies.

## Funding

This research was funded by the Hungarian National Research, Development and Innovation Office (NKFIH FK138462) (R. Kovács). R. Kovács was supported by the János Bolyai Research Scholarship of the Hungarian Academy of Sciences. R. Kovács was supported by the ÚNKP‐21‐5 New National Excellence Program of the Ministry for Innovation and Technology from the Source of the National Research, Development and Innovation Fund.

## Conflict of Interest

L. Majoros received conference travel grants from MSD, Astellas and Pfizer. R. Kovács declares no conflicts of interest.

## Author Contributions

Conceptualization, methodology and writing were performed by R.K. and L.M.
